# Numerical factorization of a matrix-function with exponential factors in an anti-plane problem for a crack with process zone

**DOI:** 10.1098/rsta.2019.0109

**Published:** 2019-09-02

**Authors:** P. Livasov, G. Mishuris

**Affiliations:** Department of Mathematics, IMAPS, Aberystwyth University, Aberystwyth, Ceredigion SY23 3BZ, UK

**Keywords:** process zone, fracture, Wiener–Hopf method, matrix factorization

## Abstract

In this paper, we consider an interface mode III crack with a process zone located in front of the fracture tip. The zone is described by imperfect transmission conditions. After application of the Fourier transform, the original problem is reduced to a vectorial Wiener–Hopf equation whose kernel contains oscillatory factors. We perform the factorization numerically using an iterative algorithm and discuss convergence of the method depending on the problem parameters. In the analysis of the solution, special attention is paid to its behaviour near both ends of the process zone. Qualitative analysis was performed to determine admissible values of the process zone's length for which equilibrium cracks exist.

This article is part of the theme issue ‘Modelling of dynamic phenomena and localization in structured media (part 1)’.

## 1. Introduction

The theory of brittle fracture, developed by Griffith using the energy balance approach [1] and later reformulated by Irwin in terms of stress fields, implies the existence of a stress singularity at the tip of a sharp crack. It is also widely recognized that, during the fracture process, phenomena occurring close to the crack tip are closely connected to the microstructure of the material. Here, we recall the models of Irwin [2] and Orowan [3] that account for plastic behaviour near the crack tip. In [4], Neuber investigates the mechanisms of physically nonlinear stress concentrations by replacing the sharp crack with a blunt notch. Barenblatt introduced the cohesive zone model in [5,6], describing the resisting forces that occur when material elements are being pulled apart. This approach allows the elimination of the stress singularity at the crack tip, which is useful for classic finite-element modelling [7]. Cohesive zone models have been widely used to study many physical phenomena, such as crack growth in viscoelastic materials [8], debonding in composite materials [9], the fracture of adhesive joints [10], among others.

All models of fracture belong to the class of mixed boundary value problems which, in turn, can be reduced to Wiener–Hopf functional equations [11–15]. This applies to modelling cracks through a formulation that is continuous, for both static and steady-state approaches [16,17] and for dynamic problems of fractures propagating within a discrete structure. The seminal contribution made by Slepyan to modelling numerous fracture problems should be mentioned. Among others, he has developed a unique technique for tackling dynamic problems in discrete structures [16,18]. This method is extremely effective for considering both lattice structures composed of masses and connecting springs [19], and structures made of masses and beams [20,21]. The developed technique allows one to determine the fundamental properties of the solution, in particular those related to the nature of crack propagation or phase transitions, and to apply this knowledge to a wide array of applications [22]. However, most of the problems solved so far only use a scalar Wiener–Hopf equation. This is because more advanced applications result in coupled systems, taking the form of a vectorial Wiener–Hopf problem with matrix kernels [23,24]. The same issue of complicated boundary value problems leading to vectorial Wiener–Hopf-type equations is also encountered during the study of certain classes of contact problems [25,26].

The issue of multiplicative decomposition, or factorization, is one of the main stages of the Wiener–Hopf method. In the case of a scalar problem, the solution can be found explicitly [27]. For higher-order problems, however, constructive solutions are only known for specific classes of matrices. Notable techniques include efficient approximation using rational functions [28–30], while numerical factorization of generalized Khrapkov–Daniele matrices is discussed in [24]. A comprehensive review of matrix factorization techniques can be found in [31].

In this paper, we analyse the static loading of a semi-infinite interface crack between two dissimilar elastic materials. We consider a formulation where the process zone reflects the bridging effect along a finite part of the interface in front of the crack. The contact in this zone is modelled by the so-called weak imperfect interface [32] which describes soft thin adhesive joints [33]. The Wiener–Hopf kernel corresponding to this problem is a matrix-valued function containing oscillating terms. There are several approaches to matrix factorization available in this case (see [34–37]); however, for this particular problem the most appropriate is that proposed in [38]. We discuss the numerical algorithm's peculiarities and demonstrate its efficiency. Finally, we determine all fracture mechanics parameters, evaluate a condition for the existence of an equilibrium state of the crack under remote loading and compute the corresponding length of the process zone.

## 2. Problem statement

Let us consider an infinite plane occupied by two different linearly elastic and isotropic materials with shear moduli *μ*_*j*_, *j* = 1, 2. These materials are joined along a linear interface. We introduce the coordinate system (*x*, *y*), where the *x*-axis is directed along that interface, the *y*-axis is directed towards the first material and the origin is located at the point that separates the intact and damaged regions. On the interface *y* = 0, we place the process zone, −*L* < *x* < 0, which separates the cracked area, *x* < − *L*, from the region of ideal contact between the materials, *x* > 0, which is ahead of the crack tip. Here, *L* is the length of the process zone mentioned above. An external out-of-plane load *p*_*j*_(*x*) is applied at the crack faces, between some predefined points *x* = − *b* and *x* = − *a*, with *a*, *b* > 0, such that it is self-balanced in terms of the principal force

2.1 $$\int_{-\infty }^{-L}p_{1}(x)\;dx=\int_{-\infty }^{-L}p_{2}(x)\;dx.$$

**Figure 1.**
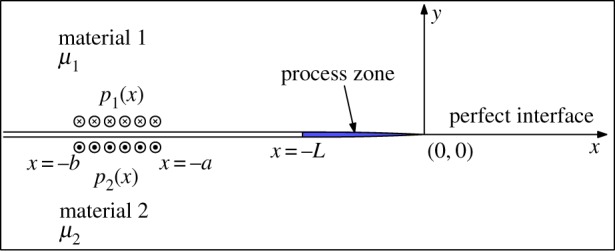
Mode III crack with process zone. (Online version in colour.)

The problem formulation outlined above is shown in figure 1.

### (a) Governing equations and boundary conditions

The equilibrium condition takes the form of the Laplace equation

2.2 $$\Delta u_{j}=0,$$

where *u*_*j*_ = *u*_*j*_(*x*, *y*) is the out-of-plane component of the displacement in the *j*th material. From [32], we have that the transmission conditions in the process zone *y* = 0,  − *L* < *x* < 0 are given by the following relations:

2.3 $$\begin{array}{rl} & \left[[u]](x,0\right)=u_{1}(x,0^{+})-u_{2}(x,0^{-})=k\sigma_{yz}^{\left(1\right)}(x,0)\\ \;\mathrm{and}\; & [[\sigma_{yz}]](x,0)=\sigma_{yz}^{\left(1\right)}(x,0^{+})-\sigma_{yz}^{\left(2\right)}(x,0^{-})=0, \end{array}\}$$

where *σ*^(*j*)^_*yz*_(*x*, *y*) = *μ*_*j*_(∂*u*_*j*_/∂*y*)(*x*, *y*) is the shear stress in the *j*th material and *k* is the interface parameter (which is similar to a spring constant [39]). Note that, in contrast to Barenblatt's model where the cohesive forces are predefined *a priori* while the length of the respective zone is computed to eliminate the stress tip singularity, our model defines only a relationship between the jump of displacements, [[*u*]], and traction, *σ*^(1)^_*yz*_, along the interface. Meanwhile, the length of the process zone is determined from additional fracture conditions related to the strength of this zone.

Along the ideal contact region, *y* = 0, *x* > 0, there is a continuity of displacements and tractions. At the crack surface *y* = 0, *x* < − *L*, we have the applied external load *σ*^(*j*)^_*yz*_(*x*, 0) = *p*_*j*_(*x*). In summary, we have the following set of boundary conditions over the whole interface *y* = 0

2.4 $$\begin{array}{rl} & \left[[u]\right](x,0)=0,\;x>0,\\ & [[u]](x,0)=k\mu_{1}\frac{\partial u_{1}}{\partial y}(x,0),\;-L<x<0,\\ & [[\sigma_{yz}]](x,0)=0,\;x>-L\\ \;\mathrm{and}\; & \sigma_{yz}^{\left(j\right)}(x,0)=p_{j}(x),\;x<-L,\;j=1,2. \end{array}\}$$

It is convenient to introduce new functions *W*,  *V*,  *Φ*,  *Ψ* and *g*_*j*_

2.5 $$\begin{array}{rl} & \left[[u]](x,0\right)=\left\{ \begin{array}{ll} 0, & x\geq 0\\ W(x), & x<0 \end{array}\right.,\;\sigma_{yz}^{\left(1\right)}(x,0)=\left\{ \begin{array}{ll} V(x), & x\geq -L\\ p_{1}(x), & x<-L \end{array}\right.,\\ & \Phi (x)=W(x-L),\;x<L,\\ & \Psi (x)=V(x-L),\;x>0\\ \;\mathrm{and}\; & g_{j}(x)=p_{j}(x-L),\;x<0,\;j=1,2. \end{array}\}$$

As a result, relations (2.4)_1_, (2.4)_3_ and (2.4)_4_ can be rewritten in the form

2.6 $$\begin{array}{rl} & \left[[u]\right](x,0)=W(x)(1-H(x)),\\ & \left[[\sigma_{yz}]](x,0)=[[p]](x)(1-H(x+L)\right)\\ \;\mathrm{and}\; & \sigma_{yz}^{\left(1\right)}(x,0)=V(x)H(x+L)+p_{1}(x)(1-H(x+L)), \end{array}\}\;|x|<\infty ,$$

where *H*(*x*) is the Heaviside function.

Following a similar approach to that outlined in [40], we look for a solution with the following asymptotic behaviour of the displacements and tractions

2.7 $$\begin{array}{rl} W(x) & =\left\{ \begin{array}{ll} U_{r}{\left(-x\right)}^{1/2}+O(x), & x\to 0^{-},\\ U_{l}+O((x+L)\ln(x+L)), & x\to -L+0,\\ O(x^{-1/2}), & x\to -\infty \end{array}\right.\\ \;\mathrm{and}\;V(x) & =\left\{ \begin{array}{ll} \sigma_{l}+O((x+L)\ln(x+L)), & x\to -L+0,\\ \tau_{r}{\left(-x\right)}^{1/2}+O(x), & x\to 0^{-},\\ \sigma_{0}x^{-1/2}+O(1), & x\to 0^{+},\\ O\left(x^{-3/2}\right), & x\to +\infty , \end{array}\right. \end{array}\}$$

where *U*_*l*_, *U*_*r*_, *σ*_*l*_, *τ*_*r*_, *σ*_0_ are some unknown constants. In the following, it will be shown that conditions above lead to a unique and physically justified solution. From (2.7), using the notation introduced in (2.5), we obtain

2.8 $$\begin{array}{rl} \Phi (x) & =\left\{ \begin{array}{ll} U_{r}{\left(L-x\right)}^{1/2}+O(L-x), & x\to L-0,\\ U_{l}+O(x\ln(x)), & x\to 0^{+},\\ O(x^{-1/2}), & x\to -\infty \end{array}\right.\\ \;\mathrm{and}\;\Psi (x) & =\left\{ \begin{array}{ll} \sigma_{l}+O(x\ln(x)), & x\to 0^{+},\\ \tau_{r}{\left(L-x\right)}^{1/2}+O(L-x), & x\to L-0,\\ \sigma_{0}{\left(x-L\right)}^{-1/2}+O(1), & x\to L+0,\\ O(x^{-3/2}), & x\to +\infty . \end{array}\right. \end{array}\}$$

The problem is now formulated in terms of the equilibrium equation (2.2) and boundary conditions (2.4)_2_, (2.6). For further analysis, it is also important to take into account the asymptotic relations (2.7) and (2.8). We note, according to (2.3), that *U*_*l*_ = *kσ*_*l*_ and *U*_*r*_ = *kσ*_*r*_.

### (b) Reduction to a vectorial Wiener–Hopf problem

To simplify later analysis, it is convenient to introduce the following constants:

2.9 $$\xi =\frac{\mu_{1}+\mu_{2}}{\mu_{1}\mu_{2}},\;\eta =\frac{\mu_{2}-\mu_{1}}{\mu_{1}\mu_{2}}\;\mathrm{and}\;d=\frac{\xi }{k}.$$

We denote the one-sided Fourier transforms of the functions *W*, *V*, *Φ*, *Ψ*, *g*_*j*_ using the standard notation

2.10 W~−(t)=∫−∞0W(x) eitx dx,Φ~−(t)=∫−∞0W(x−L) eitx dx,Im(t)<0,V~+(t)=∫0+∞V(x) eitx dx,Ψ~+(t)=∫0+∞V(x−L) eitx dx,Im(t)>0,g~j−(t)=∫−∞0pj(x−L) eitx dx,Im(t)<0, (j=1,2).}

Applying the Fourier transform to (2.2) and (2.4)–(2.6), with respect to the variable *x*, we find that the image of displacement is given by the following expression:

2.11 u~j(t,y)=((−1)j−11μjξW~−(t)−e−itLμ1+μ2[[g~]]−(t)|t|)e(−1)j|t|y,Im(t)=0, j=1,2,

where ${\tilde{W}}^{-}(t)$ is unknown.

We introduce new functions *P*^−^, *G*, *F*, related to the applied load *p*(*x* − *L*):

2.12 $$P^{-}(t)=\frac{\eta }{2}{\left[[\tilde{g}]\right]}^{-}(t)+{\left\langle \tilde{g}\right\rangle }^{-}(t),\;G(t)=P^{-}(t)-\frac{P^{-}(0)}{1-it\gamma }\;e^{itL}\;\mathrm{and}\;F(t)=-\frac{G(t)}{\sqrt{0-it}}\;e^{-itL},$$

alongside new unknown functions *Z*^+^, *R*^−^:

2.13 $$Z^{+}(t)=\frac{1}{\sqrt{0-it}}\left[{\tilde{\Psi }}^{+}(t)+\frac{P^{-}(0)}{1-it\gamma }\;e^{itL}\right]\;\mathrm{and}\;R^{-}(t)=\sqrt{0+it}{\tilde{W}}^{-}(t).$$

In (2.10)–(2.13), the tildes (∼) correspond to Fourier transforms, while the ‘ ± ’ superscript denotes that the transform is regular when ± *Im*(*t*) > 0. Here ${\left[[\tilde{g}]\right]}^{-}={\tilde{g}}_{1}^{-}-{\tilde{g}}_{2}^{-}$ is called a jump, and ${\left\langle \tilde{g}\right\rangle }^{-}=({\tilde{g}}_{1}^{-}+{\tilde{g}}_{2}^{-})/2$ is the average. Note that the auxiliary parameter *γ* is introduced with the sole limitation that *Re*(*γ*) > 0, in order to eliminate the possible singularity of the auxiliary function *Z*^+^(*t*) at zero.

The function $\sqrt{0\pm it}$ is regular in the half-plane ∓*Im*(*t*) > 0 and has a branch cut {*t* | *Re*(*t*) = 0,  ± *Im*(*t*) > 0}. It is also important to mention that the asymptotic behaviour of $R^{-},{\tilde{\Phi }}^{-},{\tilde{V}}^{+},Z^{+}$ can be obtained from (2.7) and (2.8) by means of Abel-type theorems [11]. Indeed, functions $R^{-},{\tilde{\Phi }}^{-},Z^{+}$ decay as *t*^−1^ at infinity, while ${\tilde{V}}^{+}$ decays like *t*^−1/2^ (in the corresponding half-planes). At the zero point, *t* = 0, we find that the functions $R^{-},{\tilde{V}}^{+},Z^{+}$ are bounded, while function ${\tilde{\Phi }}^{-}$ has a singularity of order *t*^−1/2^.

In addition, the original problem transforms to a matrix Wiener–Hopf equation along the real axis:

2.14 $$w^{-}(t)+M_{L}(t)v^{+}(t)=f_{L}(t),$$

where

2.15 $$w^{-}(t)=\frac{1}{\xi }\left(\begin{array}{c} {\tilde{\Phi }}^{-}(t)\\ R^{-}(t) \end{array}\right),\;v^{+}(t)=\left(\begin{array}{c} Z^{+}(t)\\ {\tilde{V}}^{+}(t) \end{array}\right)\;\mathrm{and}\;f_{L}(t)=\left(\begin{array}{c} -\frac{G(t)}{|t|}+\frac{1}{d}\frac{P^{-}(0)}{1-it\gamma }\;e^{itL}\\ F(t) \end{array}\right),$$

and

2.16 $$M_{L}(t)=\left(\begin{array}{cc} \frac{1}{\sqrt{0+it}}+\frac{1}{d}\sqrt{0-it} & -\frac{1}{d}\;e^{itL}\\ e^{-itL} & 0 \end{array}\right).$$

The total index of this matrix, *κ* = *κ*_1_ + *κ*_2_, is zero since *det* **M**_*L*_(*t*) = const [41]. In general, partial indices *κ*_1_, *κ*_2_ may be non-zero, which affects the uniqueness of the solution and requires the fulfilment of additional conditions for the function **f**_*L*_(*t*). The two simplest cases *L* = 0 (see [42]) and *L* → ∞ (for example, [32,40]) both yield a unique solution. Therefore, one can expect that matrix **M**_*L*_(*t*) will admit a stable factorization for an arbitrary finite *L*≠0.

## 3. Solution of the matrix Wiener–Hopf equation

Let us introduce function *D*(*t*) defined on the real axis

3.1 $$D(t)=\frac{d+|t|}{\sqrt{0+it}},$$

and perform both the additive and multiplicative splittings

3.2 $$D(t)=K^{+}(t)+K^{-}(t)\;\mathrm{and}\;D(t)=T^{+}(t)T^{-}(t),$$

where

3.3 $$\begin{array}{rl} K^{+}(t) & =\sqrt{0-it},\;K^{-}(t)=\frac{d}{\sqrt{0+it}},\;T^{+}(t)=\frac{\sqrt{0-it}}{Q^{+}(t)},\;T^{-}(t)=\frac{1}{Q^{-}(t)}\\ \;\mathrm{and}\;Q^{\pm }(t) & =\exp\left\{\pm \frac{1}{2\pi i}\left({\mathrm{Li}}_{2}^{\pm }\left(1+\frac{d}{t}\right)-{\mathrm{Li}}_{2}^{\pm }\left(1-\frac{d}{t}\right)\right)\right\}, \end{array}\}$$

and

3.4 Li2±(1−dt)=Re(Li2(1−dt))−i Im(ln∓(1−dt))ln±dtandLi2±(1+dt)=Re(Li2(1+dt))−i Im(ln±(1+dt))ln∓(−dt).}

Here ln_ ± _(*t*) is a branch of *log* *t* along the imaginary axis to be regular in the half-plane ± *Im*(*t*) > 0 and *Li*_2_(*z*) is the dilogarithm, defined by (see for example [43])

3.5 $${\mathrm{Li}}_{2}(z)=-\int_{0}^{z}\ln\left(1-x\right)\frac{dx}{x},\;z\in C\setminus [1,\infty ).$$

According to [43], the imaginary part of the dilogarithm *Li*_2_(1 − *t*) has a jump across the negative half of the real axis *Im*(*t*) = 0 while the real part is continuous in the entire complex plane.

In addition to this, we observe that *Q*^ ± ^(*t*)∼1 at infinity, while it decays as $\sqrt{t}$ when *t* approaches zero from the corresponding half-plane. Similarly, functions *Q*^ ± ^(*t*) in (3.3)_2_ give a closed form factorization of the function *Q*(*t*) = |*t*|(*d* + |*t*|)^−1^, representing the Wiener–Hopf kernel of the equation (2.14) in the case *L* → ∞. It is worth noting that the case of an infinite plane with a semi-infinite interfacial crack was considered in [33], where a function similar to 1/*Q*(*t*) was factorized numerically.

### (a) Iterative procedure

Following the approach outlined in [38], we approximate *Z*^+^, *R*^−^ (2.13) by functions *Z*^+^_n_*__, *R*^−^_n_*__, which are determined using the recurrence relations

3.6 Zn+(t)=1T+(t)(q+(t)−eitL((1/ξ)K+Rn−1−)+T−(t))+,Im(t)>0andRn−(t)=ξT−(t)(f(t)+e−itL[q−(t)−(K−Zn+)−]T+(t))−,Im(t)<0,}

where $n=\bar{1,n_{*}}$ and

3.7 $$f(t)=-P^{-}(t)\;e^{-itL}\;\mathrm{and}\;q(t)=-d\frac{G(t)}{\sqrt{0-it}\sqrt{0+it}}+\frac{P^{-}(0)}{1-it\gamma }\;e^{itL}.$$

The iterations start with the initial condition

3.8 $$R_{0}^{-}(t)\equiv 0,$$

and n_*_ is the required number of iterations. Note that in (3.6) the functions inside the large brackets are continuous along the real axis, while they decay at infinity as *t*^−1/2^ and *t*^−1^ for (3.6)_1_ and (3.6)_2_, respectively. The convergence of this procedure has been discussed in [38]. As soon as *Z*^+^ and *R*^−^ are evaluated, the calculation of ${\tilde{V}}^{+}$ and ${\tilde{\Phi }}^{-}$ becomes straightforward. Indeed, from (2.14) we derive a scalar Wiener–Hopf equation with respect to the unknown functions ${\tilde{\Phi }}^{-}$, ${\tilde{V}}^{+}$:

3.9 1ξΦ~−(t)−1d eitLV~+(t)=G(t)|t|+1dP−(0)1−itγ−1dD(t)Zn+(t),Im(t)=0,

which can be solved analytically. In general, for a given function *h*(*t*) that decays with the growth of |*t*|, the decomposition *h*(*t*) = *h*^+^(*t*) + *h*^−^(*t*) is accomplished by means of Cauchy-type integrals. Their limiting values along the real axis are defined by the Sokhotsky–Plemelj formulae [27]

3.10 h±(t)=h(t)2±12πi p.v. ∫−∞∞h(x)x−t dx,Im(t)=0.

### (b) Convergence

To provide an illustrative numerical example, we consider a symmetric uniform loading of unit magnitude, distributed along the line segment ( − *b*, − *a*) (figure 1). We normalize the damage zone length by a parameter l_*_ = 10(*b* − *a*) such that

3.11 $$L_{*}=\frac{L}{l_{*}}$$

is a dimensionless parameter.

**Figure 2.**
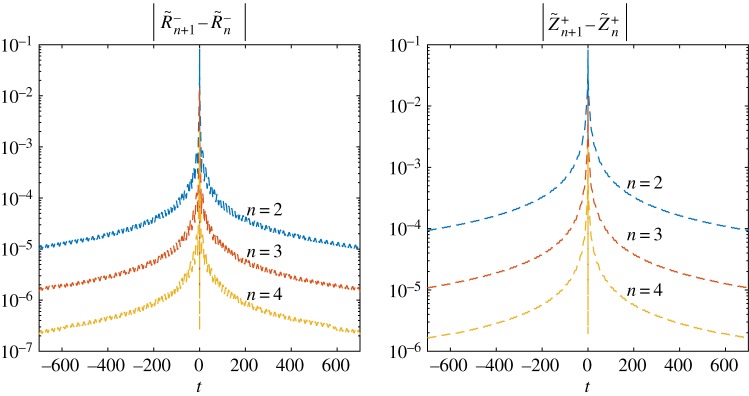
Reduction of the error with growing number of iterations for L_*_ = 0.5. (Online version in colour.)

For this formulation, the distributions of errors |*R*^−^_*n*+1_(*t*) − *R*^−^_*n*_(*t*)|, |*Z*^+^_*n*+1_(*t*) − *Z*^+^_*n*_(*t*)| along the real axis are shown in figure 2. It is clear that the numerical error decreases significantly as the iterative process continues. Note that the highest error occurs near *t* = 0, even though the functions *R*^−^, *Z*^+^ are bounded at the zero point. This is because the next term in their asymptotic expansion is of the order *t*^1/2^, and as such calculating the coefficients corresponding to these terms yields computational errors, which result in the noticeable inaccuracies at *t* = 0. It should be noted, however, that this error does decrease as the iterative process continues.

**Figure 3.**
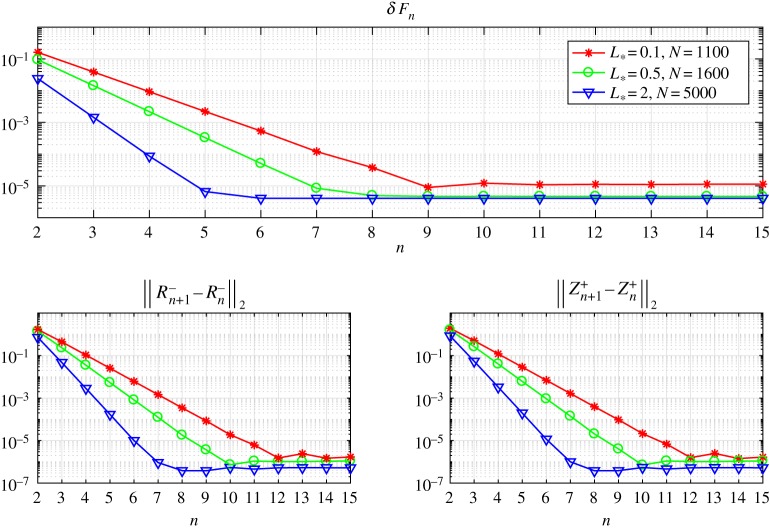
The discrepancy of the equation and the norms of errors versus the number of iterations for various numbers of integration points *N*. (Online version in colour.)

**Figure 4.**
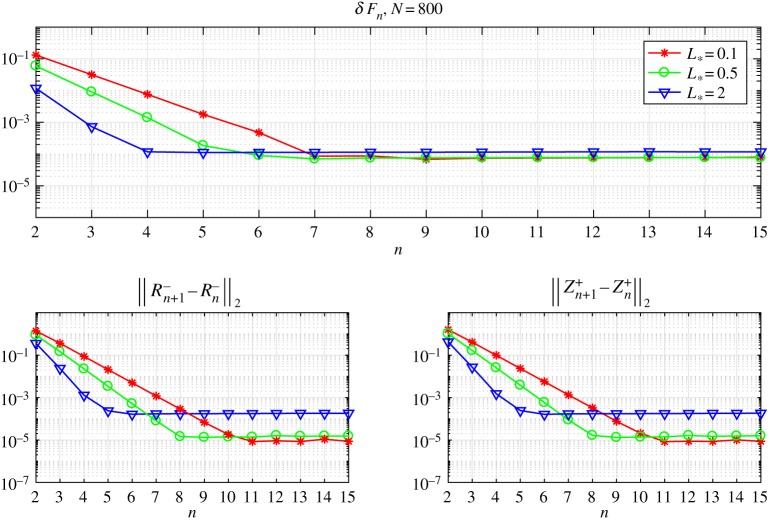
The discrepancy of the equation and the norms of errors versus the number of iterations for a fixed number of integration points *N* = 800. (Online version in colour.)

Going further, in figures 3 and 4, we show that the iterative method's rate of convergence increases with the growth of the process zone L_*_. To demonstrate this we introduce the discrepancy *δF*_*n*_, which represents the accuracy of the solution to the Wiener–Hopf equation (2.14)

3.12 $$\delta F_{n}=∥F-F_{n}{∥}_{2}\;\mathrm{and}\;F_{n}(t)=\frac{1}{\xi }R_{n}^{-}(t)+e^{-itL}Z_{n}^{+}(t),$$

where *F*_*n*_ is the left-hand side of the second component of the vectorial Wiener–Hopf equation (2.14), while *F* is given in (2.12). In (3.12) and below, we denote the $L_{2}$ norm on R by ∥ · ∥_2_.

Additionally, in these figures, we also show the distributions of three measures: the quantity *δF*_*n*_ mentioned above, and the norms of difference between the consecutive iterative steps ∥*R*^−^_*n*+1_ − *R*^−^_*n*_∥_2_, ∥*Z*^+^_*n*+1_ − *Z*^+^_*n*_∥_2_, to provide a fuller picture of the numerical method's rate of convergence.

One can observe in figure 3 that the iterative method (3.6) converges faster (in terms of the number of iterations) and gives more accurate results for higher values of L_*_. However, it is important to account for the fact that functions *Z*^+^(*t*) and *R*^−^(*t*) oscillate with frequencies that grow as we increase L_*_ and, consequently, a larger number of mesh nodes *N* are needed to account for this. This, in turn, makes each individual iteration more time-consuming.

To further clarify this point, and the dependence of the final algorithm on the number of integration points *N*, in figure 4 the same graphs are provided for fixed *N* = 800 with differing values of L_*_. It is clear that the number of iterations needed to reach the saturation limit decreases with increasing L_*_. On the other hand, for a fixed value of *N*, the numerical method gives more accurate results for smaller values of L_*_. The latter issue is eliminated by taking the number of mesh nodes *N* as a function of L_*_, as demonstrated by the results in figure 3.

It is worth noting that calculating the functions *Z*^+^_*n*_, *R*^−^_*n*_ at each step of the iterative process (3.6) only involves evaluating the singular integrals within (3.10), while *K*^ ± ^ and *T*^ ± ^ remain the same for every iteration step. The integration in (3.6) was performed numerically on a finite part of the real axis, while taking into account the asymptotic behaviour of the integrand at infinity. Finally, to increase the algorithm's efficiency, the integration interval was partitioned non-uniformly.

In table 1, we show the time costs for different computational scenarios. The calculations were performed on a computer with an Intel Core i3-2310M CPU @ 2.10 GHz  ×  2 processor.

**Table 1.** Time costs.

	*N* = 800			*N* = 1100	*N* = 1600	*N* = 5000
	L_*_ = 0.1	L_*_ = 0.5	L_*_ = 2	L_*_ = 0.1	L_*_ = 0.5	L_*_ = 2
number of iterations before saturation	11	8	6	12	10	8
average time per iteration (s)	29	29	29	50	70	320
total time (s)	330	249	195	650	784	2685
error *δF*_*n*_	7 × 10^−5^	7 × 10^−5^	10^−4^	10^−5^	5 × 10^−6^	4 × 10^−6^

## 4. Analysis of the numerical results

With the iterative scheme for solving the vectorial Wiener–Hopf problem in place, and the accuracy of the numerical algorithm demonstrated, we can begin the evaluation and analysis of the system behaviour. We begin with an examination of the fracture mechanics parameters, before moving on to the process zone itself, and finally determining the fracture's equilibrium conditions.

### (a) Evaluation of the fracture mechanics parameters

The known asymptotics of functions *W*, *Φ*, *V*, *Ψ* in the limits as *x* → 0^−^ and *x* →  − *L* (2.7)–(2.8) allow us to define the behaviour of the stresses and displacements at both ends of the process zone. For example, the behaviour of the stress functions as *x* → 0^+^ follows immediately from (2.6) as: $\sigma_{yz}^{\left(j\right)}(x,0)∼\sigma_{0}/\sqrt{x}$. Noting this, linear elastic fracture mechanics (LEFM) allows us to immediately introduce the stress intensity factor

4.1 $$K_{\mathrm{III}}^{L}=\sqrt{2\pi }\sigma_{0},$$

and analyse its dimensionless value *K*^*L*^_*III*_/*K*_*III*_, where *K*_*III*_ is the stress intensity factor in the absence of the damage zone (*L* = 0), taken from [42]. Next, we evaluate the parameter *σ*_*l*_, that is the stress level at the left edge of the process zone (2.7). Then, combining the relationship between the jump of displacements and traction (2.3) in this zone with the known asymptotics yields *U*_*l*_ = *kσ*_*l*_, from which we can obtain the displacement of the crack opening at *x* = − *L* in its dimensionless form

4.2 $$u_{l}^{*}=2\frac{k}{\xi }\frac{\sigma_{l}}{\sqrt{l_{*}}K_{\mathrm{III}}}.$$

**Figure 5.**
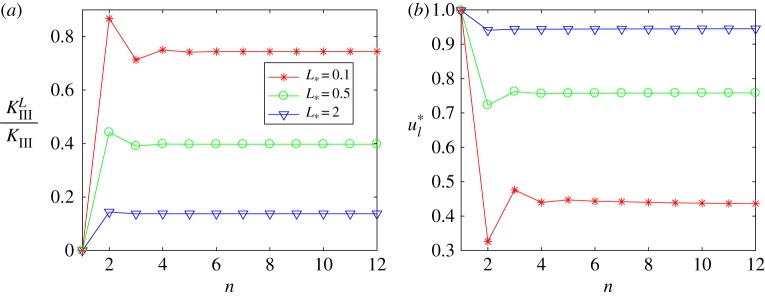
Convergence of the values of LEFM parameters with the iterations. (Online version in colour.)

The rates at which the values of *K*^*L*^_*III*_/*K*_*III*_ and u*_l_ stabilize throughout the iterative process for various *L* are provided in figure 5.

Meanwhile, to demonstrate the numerical error in approximating the fracture parameters, we introduce the following measures

4.3 $$\delta K_{\mathrm{III}}^{\left(n\right)}=\left|\frac{K_{\mathrm{III}}^{L}(n+1)}{K_{\mathrm{III}}}-\frac{K_{\mathrm{III}}^{L}(n)}{K_{\mathrm{III}}}\right|\;\mathrm{and}\;\delta u_{l}^{\left(n\right)}=|u_{l}^{*}(n+1)-u_{l}^{*}(n)|.$$

**Figure 6.**
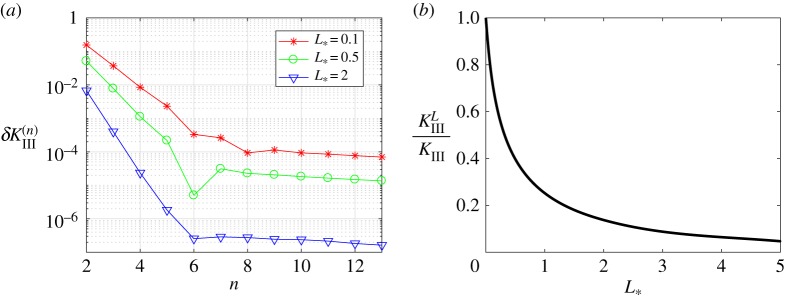
Normalized stress intensity factor. (Online version in colour.)

**Figure 7.**
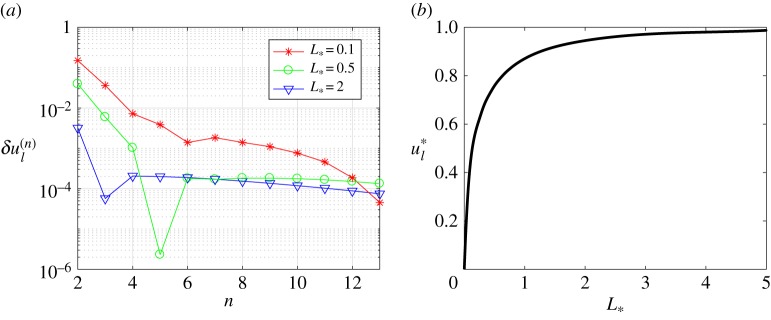
Normalized crack opening at *x* = − *L*. (Online version in colour.)

The rate at which these errors decrease throughout the iterative process, alongside the dependence of the fracture parameters on the length of the process zone, are shown in figures 6 and 7. It should be noted that *K*^*L*^_*III*_/*K*_*III*_ = 1 and u*_l_ = 0 when *L* = 0.

It can easily be seen in figures 5–7 that the outlined method is able to achieve a high accuracy of approximation for the stress intensity factor and crack opening displacement. This is achieved after only a small number of iterations, and with a high level of stability of the numerical algorithm.

Note that figures 6 and 7 establish implicit relationships between the length of the process zone and two fracture parameters under consideration. They will be used later to identify admissible length of the process zone for a stable crack, see §4c.

### (b) Displacements inside the fracture zone

To conduct an examination of the process zone, *y* = 0, − *L* < *x* < 0, we recall from (2.6) that *W*(*x*) corresponds to the jump of displacements along the interface [[*u*]](*x*, 0) = *W*(*x*)(1 − *H*(*x*)). In fact, the displacements *u*_*j*_(*x*, 0), (*j* = 1, 2) can be obtained in terms of this function by applying the inverse Fourier transform to (2.11), while for the traction we simply use the transmission conditions (2.3)

4.4 $$u_{j}(x,0)={\left(-1\right)}^{j-1}\frac{1}{\xi \mu_{j}}W(x),\;\sigma_{yz}^{j}(x,0)=\frac{1}{k}W(x),\;-L<x<0.$$

To demonstrate the solution convergence for the function *W*(*x*), we consider the case^1^ when L_*_ = 0.5. Similarly to the previous subsection, we introduce the new measure

**Figure 8.**
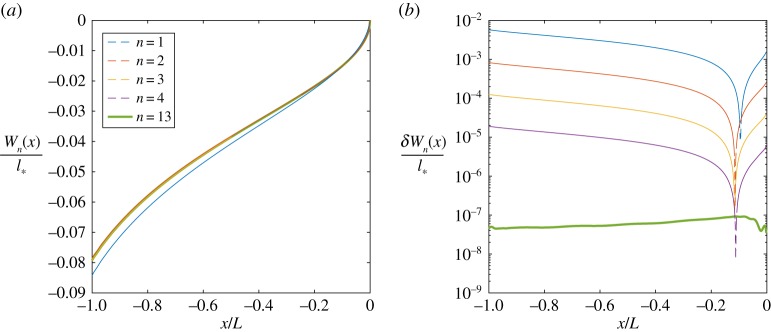
Convergence of *W*_*n*_(*x*) for L_*_ = 0.5.

4.5 $$\delta W_{n}(x)=|W_{n+1}(x)-W_{n}(x)|,$$

where *W*_*n*_(*x*) is the function *W*(*x*) at the *n*th iteration. The values of this measure, alongside the corresponding dimensionless solution for *W*_*n*_(*x*)/l_*_ along the process zone, are provided in figure 8. It can be clearly seen that after the second iteration the curves *W*_*n*_(*x*) almost coincide.

**Figure 9.**
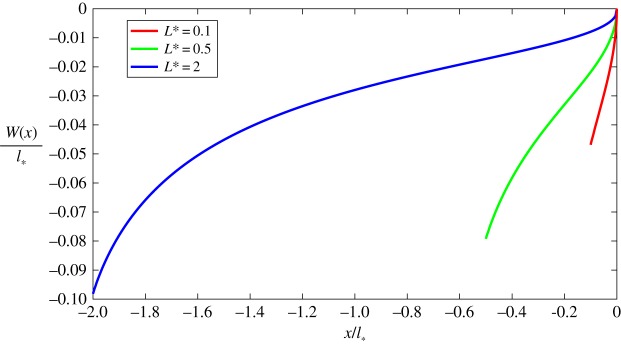
Jump of displacements for various L_*_.

With the rapid convergence of *W*_*n*_(*x*) established, we approximate the function *W*(*x*) with *W*_n_*__(*x*), where n_*_ is the number of required iterations to achieve a desired level of accuracy. Using this approach, the solution for function *W*(*x*), for different values of L_*_, are depicted in figure 9.

Therefore, as we can see from (4.4), an understanding of the system's behaviour within the process zone can be obtained simply from the analysis of *W*(*x*). In particular, one can observe, that both the displacement's discontinuity and the shear stress increase in the direction from the crack tip towards the opposite end of this zone.

### (c) Equilibrium conditions

In the previous analyses, we assumed that the length of the process zone *L* was predefined, but in reality, for equilibrium cracks, there are two critical conditions which ensure that the fracture will not propagate and that neither end of the process zone will move. These conditions can be expressed in terms of the crack opening displacement and stress intensity factor as follows:

4.6 $$\left[[u]\right](-L)<U_{C}\;\mathrm{and}\;K_{\mathrm{III}}^{L}<K_{C},$$

where *U*_*C*_, *K*_*C*_ are material parameters. By normalizing (4.6), these equilibrium conditions can be placed in terms of u*_l_ and *K*^*L*^_III_/*K*_III_

4.7 $$u_{l}^{*}<U_{C}^{*},\;\frac{K_{\mathrm{III}}^{L}}{K_{\mathrm{III}}}<\frac{K_{C}}{K_{\mathrm{III}}},$$

where

4.8 $$U_{C}^{*}=\frac{2U_{C}}{\xi \sqrt{l_{*}}K_{\mathrm{III}}}.$$

**Figure 10.**
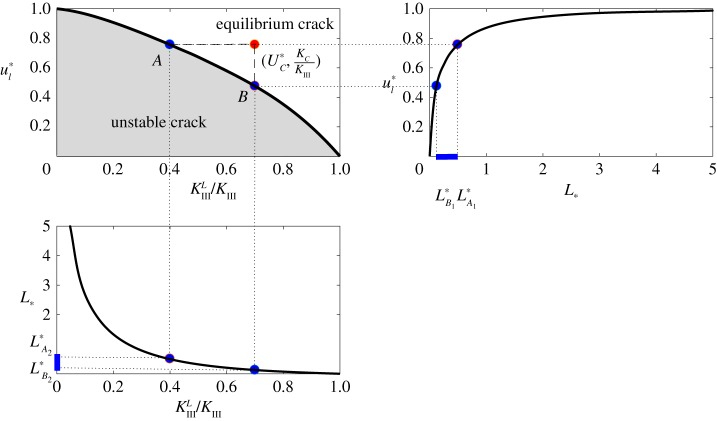
Admissible values of L_*_. (Online version in colour.)

In this form, the equilibrium conditions can be related back to the normalized cohesive zone length L_*_ using observations from §4a. Combining the results displayed in figures 6 and 7, the relationship between u*_l_ and *K*^*L*^_III_/*K*_III_ is immediately obtained, as provided in the top left of figure 10. This line represents the boundary between unstable and equilibrium cracks. Indeed, provided critical values of the crack opening displacement U*_C_ and fracture toughness *K*_*C*_/*K*_III_ are taken from the white region (see the red marker in figure 10), conditions (4.7) are automatically satisfied. From this, alongside previously obtained relations, admissible values of L_*_ for equilibrium cracks can be defined.

To better illustrate this point, consider taking two arbitrary points *A*,  *B* along the boundary between the unstable and equilibrium regimes (figure 10). Then, by definition, at the point *A* we have u*_l_ = U*_C_, while the corresponding value of L_*_ can obtained from figure 7, which we shall denote by L*_A_. Furthermore, it is clear that condition (4.7)_1_ is satisfied for any L_*_ < L*_A_. Similarly, point *B* defines the critical process zone length L*_B_, with condition (4.7)_2_ holding for any L_*_ > L*_B_. It should be noted, as seen in figure 10, that the values of (L*_B_,  L*_A_) obtained from each condition are identical

4.9 $$(L_{B_{1}}^{*},\;L_{A_{1}}^{*})=(L_{B_{2}}^{*},\;L_{A_{2}}^{*})=(L_{B}^{*},\;L_{A}^{*}).$$

To summarize, the region (L*_B_,  L*_A_) obtained using the approach outlined above defines the range of the normalized length of the damage zone, L_*_, over which the equilibrium state (4.6) is possible. Furthermore, if the critical fracture parameters for a given problem fall within the grey region in the top left of figure 10, then for any L_*_ at least one condition in (4.7) fails and the crack becomes unstable.

### (d) Impact of the damage zone's stiffness

To conclude the analysis of the given problem, we examine how the parameter of the process zone, *k*, affects other fracture parameters. This is achieved through an examination of the normalized constant d_*_, defined as

**Figure 11.**
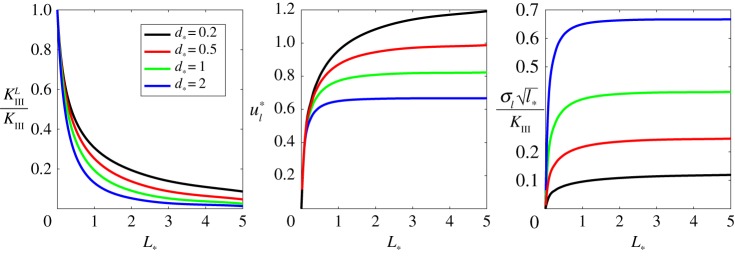
Stress intensity factor, crack opening and critical stress in the process zone for various d_*_.

4.10 $$d_{*}=\frac{l_{*}}{k}\frac{\mu_{1}+\mu_{2}}{\mu_{1}\mu_{2}}=l_{*}d,$$

where constant l_*_, which was introduced to ensure a proper scaling, was previously determined in (3.11), while the parameter *d* was introduced in (2.9). Note that all of the results presented in the previous subsections were obtained for a fixed value of d_*_ = 0.5. The relationships between the normalized process zone length and the stress intensity factor, crack opening and critical process zone stress are provided in figure 11 for varying values of the parameter d_*_.

It is readily apparent from the figure above that as the constant *k* increases so do the value of *K*^*L*^_III_ and *u*_*l*_, while the critical stress *σ*_*l*_ decays. In other words, an increase in the parameter *k* leads to a weaker response to the crack within the process zone, while for smaller *k* this zone demonstrates tougher behaviour as reflected in the growth of *σ*_*l*_ (in the right part of figure 11). Note that, in the limit as *k* → 0, the solution is regular at *x* = 0 while the crack tip and stress singularity are located at the opposite edge of the damaged zone *x* = − *L*.

## 5. Discussions and conclusion

In this paper, we considered the static loading of a mode III crack at the interface of two elastic materials, with a process zone modelled by relations (2.3). The original problem, given in terms of equation (2.2) and boundary conditions (2.4)–(2.6), was transformed to a matrix Wiener–Hopf equation (2.14). The main difficulty in solving this equation was the presence of exponential factors inside matrix **M**_*L*_.

It has been demonstrated that an iterative approach, of the kind first proposed in [38], is highly effective at resolving this Wiener–Hopf equation, thereby both solving the initial problem and determining important fracture mechanics parameters for the system. Namely, the stress intensity factor, *K*_*III*_, at the crack tip and the critical opening, *u*_*l*_, at the opposite end of the process zone. A thorough analysis of the numerical approach used showed the high level of stability and accuracy, alongside the rapid rate of convergence, for the entire solution. Of particular note was the fact that the values of both *K*_*III*_ and *u*_*l*_ stabilize after only a small number of iterations, although the required number of iterations is higher for smaller lengths of the process zone *L*.

The fact that the method outlined here is more efficient for larger *L* is not unexpected, as the initial condition (3.8) of the iterative process corresponds to the case *L* → ∞. As such, although the iterative method does converge for all positive *L* > 0, it is clear from the presented results that both the solution accuracy and convergence rate improve with the growth of *L*. There are however solutions to this issue. The computational performance, in this case, could be greatly increased through a rescaling of the *x*-axis, although this would introduce an additional challenge in the manipulation of the right-hand side of (2.14). Alternatively, for small *L* there exists an alternative asymptotic method for the matrix factorization, which was presented in [35].

In addition, it was demonstrated that the relationship between critical values of the crack opening displacement and the stress intensity factor represent a boundary between unstable and equilibrium cracks. This result was achieved through qualitative analysis, performed to determine admissible values of length of the process zone (4.9) for which both necessary conditions for equilibrium in (4.6) are satisfied.

Meanwhile, the impact of the stiffness parameter, 1/*k*, on the solution was shown to become more pronounced as *k* increases, with the crack opening growing while the traction inside the damage zone decays. Conversely, reducing *k* results in a higher resistance to the crack opening within the process zone. Indeed, for a significantly stiff process zone (*k* → 0), the stress singularity at the right edge vanishes and a high-stress concentration appears near the point *x* = − *L*.

Finally, the model considered in this paper can be used to analyse steady-state propagation of an interface crack with a developed damage zone. Similar models with a semi-infinite crack and various imperfect material interfaces were considered in [32]. In the former problem, the crack speed can influence the size of the damage zone and consequently affect the stability of the crack propagation.

## Footnotes

1 The authors also considered the cases when *L*_*_ = 0.1 and *L*_*_ = 2 and obtained similar results, as such taking *L*_*_ = 0.5 provides a representative illustration.

## Data accessibility

This article has no additional data.
